# Therapy for Dupuytren’s Disease (II): Collagenase Therapy vs. Limited Fasciectomy—A Long-Term Comparative Study

**DOI:** 10.3390/life15010076

**Published:** 2025-01-10

**Authors:** Nikolaus Wachtel, Francesca Romana Dingler, Constanze Kuhlmann, Sinan Mert, Elisabeth Maria Haas-Lützenberger, Verena Alt, Nicholas Moellhoff, Riccardo Giunta, Wolfram Demmer

**Affiliations:** Department of Hand, Plastic and Aesthetic Surgery, LMU Klinikum, Ziemssenstraße 5, 80336 Munich, Germany

**Keywords:** Dupuytren’s disease, palmar fascial fibromatosis, Viking disease, contracture, collagenase, Xiapex, limited fasciectomy

## Abstract

Background: Dupuytren’s disease (DD) is a systemic connective tissue disorder of the palm, predominantly affecting men of Northern European or Caucasian origin over 55. In addition to conventional surgery, Dupuytren’s contracture can be treated in a minimally invasive way by injecting bacterial collagenase into the cord. However, studies on the long-term success rate when compared to the gold standard, surgical limited fasciectomy, are limited. Methods: This monocentric retrospective study examined 35 patients who had been treated with bacterial collagenase for Dupuytren’s contracture, conducting a long-term follow-up after an average of 5.7 years. The results were compared to a control group of 40 patients treated with surgical limited fasciectomy on average 5.5 years ago. Finger extension (Tubiana stage), strength, sensitivity, the effect of possible risk factors, and patient-reported outcome measures (PROMs) were compared between the two groups. Results: The long-term results after therapy for DD showed a significant reduction in the Tubiana stage for both groups (*p* < 0.001). Additionally, we observed a longer mean preintervention Tubiana stage and a better long-term improvement in the Tubiana stage for patients with limited fasciectomy when compared to the collagenase group. (both *p* < 0.001). Neither grip strength nor the pinch test showed significant differences when compared within each group or when comparing both groups. Both the treated and untreated fingers of patients with limited fasciectomy had a superior two-point discrimination (*p* < 0.001). For the URAM questionnaire, we observed a significantly better result in the control group (*p* < 0.01). Retrospectively, significantly more patients in the collagenase group would not choose the same therapy to treat DD (35 vs. 8%; *p* < 0.05). Conclusions: The two therapy options should be seen as complementary for the treatment of DD. Collagenase therapy seems a sensible option for DD with an earlier Tubiana stage and contractures that predominantly affect the MCP joint. Contractures with higher Tubiana stages that also affect the PIP joint should predominantly be treated with limited fasciectomy.

## 1. Introduction

Dupuytren’s disease (DD) is a benign, typically progressive, and painless fibrotic disorder that affects the palmar fascia of the hand [1,2,3]. It primarily affects men of Northern European or Caucasian descent, typically beginning after the age of 55 [2,4]. Women are affected less frequently and usually experience a milder progression of the disease [4,5,6,7].

Open surgical limited fasciectomy is regarded as the gold standard in the current literature for treating Dupuytren’s disease due to its ability to provide the best long-term outcomes. As a result, it is the most commonly used treatment for DD in Europe [8,9,10]. Surgical resection of the Dupuytren’s cord is particularly indicated for higher-grade contractures, involvement of multiple fingers, severe movement restrictions in the PIP and DIP joints, and in recurrence treatment [11]. During surgery, the contracture and nodular changes are partially or completely removed, and the wound is closed with skin flaps [4]. A disadvantage of this approach is the risk of complications such as wound healing problems, skin necrosis, infections, persistent numbness, cold sensitivity, and stiffness, as well as major complications such as injuries to arteries, nerves, or tendons [12,13].

In minimally invasive needle fasciotomy, palpable Dupuytren’s cords are perforated multiple times through the skin using a fine needle. By passively extending the finger until the cord breaks completely, the finger contractures are resolved [14]. Needle fasciotomy carries multiple risks, such as skin tears, tingling sensations, flexor tendon injuries, and vascular and nerve lesions, up to fractures and joint injuries [14]. Moreover, the recurrence rate for this procedure is high [12,15].

The therapy with microbial collagenase is a relatively new minimally invasive therapy for DD. The procedure involves the injection of enzymes produced by Clostridium histolyticum (Xiapex^®^, Pfizer Inc., New York, NY, USA) into Dupuytren’s cords. The Product consists of two isoforms of collagenase class I and II, AUX-I and AUX-II [16]. Both complement each other in their effects [4,16,17]. The Dupuytren’s cord, primarily composed of collagen types I and III, is hydrolytically cleaved by these enzymes [17]. Vascular and nerve structures are preserved, as the collagenases do not affect collagen type IV, the main component of the basement membrane of these structures [16,18]. Collagenase is approved for therapy for DD in adults with a palpable nodule and/or cord as well as for Peyronie’s disease (induratio penis plastica) in adult men [17]. Collagenase has been marketed under the brand name Xiapex^®^ in the European Union since 2011 as a therapy for Dupuytren’s disease. However, the product has since been withdrawn from the European market for economic reasons [17,19,20]. It continues to be marketed by Endo International under the name Xiaflex^®^ in the USA and other countries [21].

Given the lengthy recovery time and potential serious complications associated with limited fasciectomy, collagenase therapy seems to be a well-tolerated, minimally invasive alternative with lower morbidity compared to limited fasciectomy. It also has a lower recurrence rate than needle fasciotomy. In fact, we were recently able to show that collagenase therapy resulted in significant long-term improvements in contracture for both the MCP and PIP joints [22].

However, the data on the effectiveness of collagenase therapy compared to the gold standard (limited fasciotomy/fasciectomy) remains limited [23,24]. To assess the potential therapeutic benefits of each treatment approach, we analyzed the long-term success of collagenase therapy in DD, comparing it to limited fasciotomy/fasciectomy as a control group. This study focuses on changes or improvements in finger contracture, recurrence rates, and Patient-Reported Outcome Measures (PROMs). By comparing collagenase therapy to the established gold standard treatment for DD, we aim to evaluate its therapeutic benefit, considering patients’ subjective assessments.

## 2. Materials and Methods

This monocentric retrospective study was conducted at a university hospital in southern Germany with specialized hand surgery care between 2011 and 2015. All patients with DD who qualified and were willing to undergo microbial collagenase treatment or partial fasciectomy, respectively, and who consented to participate in the study, were included. Ethical approval for the study was granted by the local ethics committee (Project Number: 18-265, Date: 8 June 2018). All patients provided written confirmation of their voluntary participation in the study and were over 18 years of age. The results of the early postoperative follow-up evaluations for collagenase treatment were previously published in 2017 by Pototschnig et al. [25].

Criteria for inclusion in the collagenase group were the presence of Dupuytren’s contracture with a palpable nodule and/or cords requiring therapy, as well as the patient’s assent. Patients who had previously received surgery or treatment on the affected finger were excluded from this study. Additionally, a retrospective control group was established with patients who had undergone limited fasciectomy in our clinic between 2012 and 2015. In both groups, patients who were deceased or were seriously ill, as well as those who could not be contacted or did not agree to continue participation in the study, were excluded from the follow-up. Moreover, patients who had undergone subsequent (surgical) treatment on the affected finger during the course of the study were also excluded from follow-up (secondary exclusion).

### 2.1. Microbial Collagenase

The product used consists of microbial collagenase class I and II (AUX-I, AUX-II) derived from Clostridium histolyticum. The injection solution was prepared as per the manufacturer’s instructions by mixing 0.9 mg of collagenase powder with the solvent.

The administration and collagenase therapy followed the guidelines outlined in our previously published article: Therapy for Dupuytren’s Disease: Collagenase Therapy—A Long-Term Follow-Up [22]. An injection volume of 0.25 mL was infiltrated into cords involving the MCP joint, while 0.20 mL was infiltrated into cords over the PIP joint. The injection was administered intralesionally where the cord was furthest from the flexor tendon and, wherever possible, without adherence to the skin. On the subsequent day, the cord was ruptured through passive extension of the finger under local anesthesia with Scandicain. For this, the patient’s wrist was held in a flexed position while the treating physician applied moderate pressure on the cord by extending the finger for 10–20 s. The treatment was always performed by an experienced hand surgeon trained in this procedure. After the surgery, a palmar splint was applied to keep the fingers extended until the sutures were removed. Patients were also prescribed physiotherapy for 2 to 4 weeks and advised to wear a night splint for 4 months following the procedure [22].

### 2.2. Open Surgical Limited Fasciectomy

The procedure was performed by a fully trained hand surgeon in a standardized manner, using magnification under sterile conditions in a fully equipped operating room. During surgery, the skin and subcutaneous fat were carefully separated from the Dupuytren’s cord. During the preparation and resection of the altered fascial tissue, structures such as blood vessels, nerves, and tendons were explicitly preserved. After hemostasis, the skin was closed without tension. In cases of larger skin defects where primary wound closure was not possible, appropriate local flaps were performed. A palmar splint was used postoperatively to maintain the fingers in an extended position until the sutures were removed. Physiotherapy was prescribed starting two weeks after surgery, along with a night positioning splint for four months postoperatively.

### 2.3. Follow-Up Examination

Using a standard hand goniometer (AFH Webshop, Lügde, Germany) the active and passive range of motion (ROM) of metacarpophalangeal (MCP) and proximal interphalangeal (PIP) joints of the hand was measured. The neutral 0 method of the Association for the Study of Internal Fixation (AO) was used for documentation [26]. To improve intra- and inter-rater reliability of the tests, standardized protocols were implemented [27].

Hand strength was measured using a Jamar hand dynamometer (Saehan Corporation, Changwon-City, Republic of Korea) in kilograms (kg) [28]. Hand strength was always measured on both hands, for comparison (healthy vs. treated hand). A mean out of thre measurements per hand was recorded. Pinch strength was measured between the thumb and the opposing treated finger in a similar manner using a digital pinch gauge (Fabrication Enterprises Inc., White Plains, NY, USA) [29].

To assess the sensitivity of the treated finger and its corresponding contralateral side a two-point discrimination-test on the fingertip was used. The Discriminator by AREX (Palaiseau, France) was used with a range of 1–25 mm. The smallest distance at which a patient could differentiate two cones was recorded [30,31].

### 2.4. Questionnaires/Patient-Reported Outcome Measures (PROMs)

Long-term patient satisfaction and quality of life after collagenase therapy or limited fasciectomy, respectively, was assessed using the QuickDASH (Disabilities of the Arm, Shoulder, and Hand) short questionnaire as well as the URAM (Unité Rhumatologique des Affections de la Main) survey. The QuickDASH questionnaire is a standardized questionnaire used to evaluate physical disabilities and symptoms of the upper extremity. The questionnaire consists of 11 questions covering topics such as physical activities, impact on daily life and work, and symptoms. Each question is rated from 1 (no difficulty) to 5 (extreme difficulty or unable). Summing up the scores results in a value ranging from 0 (no disability) to 100 (maximum disability) [32,33].

The URAM questionnaire is designed to capture specific aspects of rheumatological conditions and their effects on hand function and overall well-being of the patient. Many aspects relevant for evaluating Dupuytren’s disease are covered, including questions about symptoms such as movement restriction and stiffness, functional limitations such as challenges in carrying out daily tasks, the consequences of the disease on quality of life, and the subjective treatment experiences of the patients. The URAM score can take values between 0 (= best possible score) and 45 (= worst possible score) [34,35]. Additionally, the subjective assessment of the chosen therapy was assessed (Table 1).

### 2.5. Statistical Analysis

All results were statistically analyzed using SPSS (International Business Machines Corporation, Armonk, New York, NY, USA; Version 26 and 27) and GraphPad Prism 10 (GraphPad Software, Inc., San Diego, CA, USA). For all tests, a significance level of *p* ≤ 0.05 was set as the threshold for significance. The Pearson chi-squared test was used to assess significant association between categorical variables.

For the evaluation of contracture improvements, the Wilcoxon test and the Student’s *t*-test (for differences in long term improvement) were applied. To assess differences in strength, either a paired *t*-test or a Wilcoxon test was applied depending on whether the values were normally distributed or not. Differences in sensitivity between the treated hand and the contralateral side were determined using a paired *t*-test. To calculate significant differences between retrospective therapy choices, Fisher’s exact test was used.

## 3. Results

**Figure 1:**
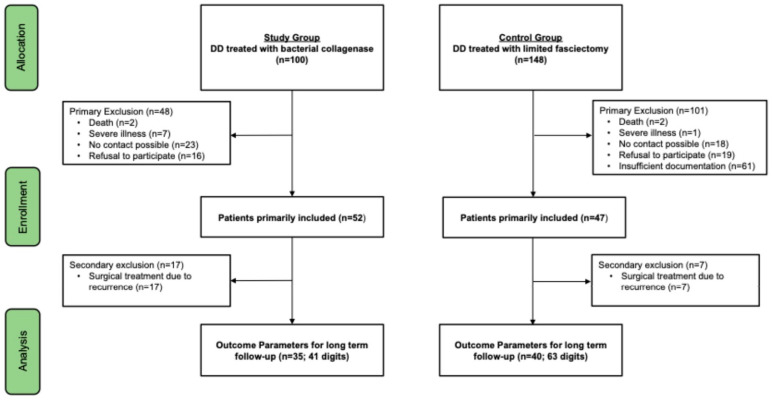
Study flow chart.

Of the initial 100 patients treated with collagenase, 52 were eligible for inclusion in the study. Of these, 17 patients received subsequent surgical treatment for DD. As a result, they were excluded from the follow-up but included in the evaluation of subjective treatment experience and postoperative compliance. This led to a drop-out rate of 48% for subjective treatment evaluation and 65% for clinical follow up. Therefore, a total of 35 patients (41 digits) were included in the long-term follow-up. Detailed results for these were previously published by our research group [22]. The control group included patients that underwent surgical limited fasciectomy for DD. Initially, 148 patients were identified that met the inclusion criteria. A total of 40 (63 digits) out of these could be included in the long-term follow-up with simultaneous PROM questionnaire evaluation. This corresponds to a drop-out rate of 73% during follow-up. The study population, including dropouts and exclusions, is illustrated in the study flow chart (Figure 1).

The mean time from collagenase treatment to follow-up was 5.7 (± 0.6 SD) years. The mean time from surgical treatment to follow-up was 5.5 (± 1.1 SD) years. In both groups, significantly more male than female patients were treated for Dupuytren’s contracture. Table 2 provides an overview of the epidemiological characteristics of the patient groups.

### 3.1. Comparison of Long-Term Outcomes of Collagenase Therapy and Limited Fasciectomy

**Figure 2:**
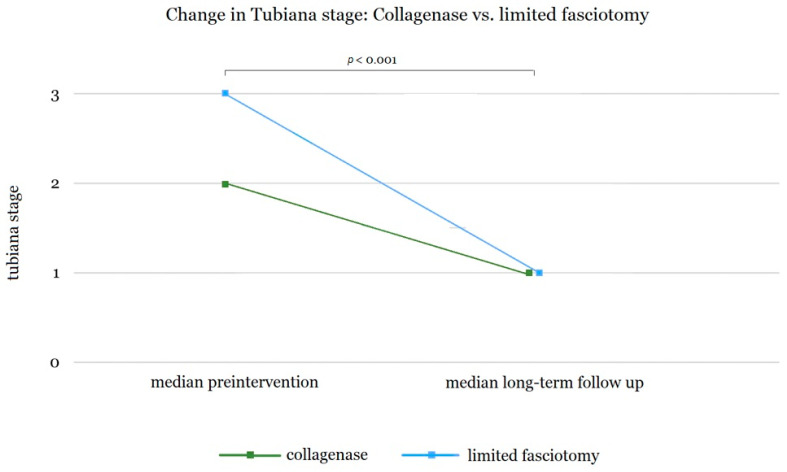
Changes in Tubiana stage in digits treated with collagenase (n = 41) and limited fasciectomy (n = 69). Comparison of preoperative and long-term follow-up after 5.5 and 5.7 years, respectively. For both groups we observed a significant reduction in the Tubiana stage in the long term follow up (both *p* < 0.001). Moreover, the control group (limited fasciectomy) demonstrated a significantly better improvement in the Tubiana stage (*p* < 0.001).

For the control group, 40 patients (63 digits) could be included for the long-term follow-up. For these, neither the individual joint nor cumulative finger contracture was properly documented; only the Tubiana stage at the time of surgery was consistently stated in the patient data. We therefore used the pre- and post-intervention Tubiana stages to compare both groups. The decrease in contracture according to Tubiana stages, both pre- and post-operatively, is shown in Figure 2.

For both groups, we observed a significant reduction in the Tubiana stage in the long-term follow-up (*p* < 0.001 for the limited fasciectomy group and *p* < 0.001 for the collagenase group). Moreover, we observed a significantly higher median preintervention Tubiana stage (3.0) for patients with limited fasciectomy when compared to the collagenase group (2.0) (*p* < 0.001). Consequently, the control group (limited fasciectomy) demonstrated significantly better improvements in the Tubiana stage (*p* < 0.001). No significant differences were found in the Tubiana stage between the two groups at the follow-up examination (median long-term follow-up for both groups: Tubiana stage 1.0).

### 3.2. Strength and Sensitivity of the Hand

For the collagenase group, paired measurements were performed 34 times for grip strength and 30 times for the pinch test. In the control group, grip strength was measured for 40 pairs, and the pinch test was performed in 58 digit pairs. For both groups, no significant difference in grip strength or the pinch test was observed in the side-by-side comparison 5.5 and 5.7 years after treatment (Table 3). Additionally, we observed no significant difference between the two groups regarding grip strength and the pinch test.

We used two-point discrimination testing to assess potential differences in sensitivity after therapy. Here, patients treated with bacterial collagenase (n = 39 digits) showed an average two-point discrimination of 4.82 mm on the treated finger and 5.15 mm on the untreated finger. In digits of patients treated with limited fasciectomy (n = 67), the closest differentiation of two-point discrimination occurred at 3.57 mm on the operated hand and 3.58 mm on the untreated hand. The difference between treated and untreated hands was not significant in either group. However, we observed a significant difference when comparing results for two-point discrimination between the two groups. Both the treated and untreated fingers of patients with limited fasciectomy had a superior two-point discrimination than digits of patients in the collagenase group (*p* < 0.001).

### 3.3. Patient-Reported Outcome Measures (PROMs)

Quality of life and the outcome were assessed with QuickDASH and URAM questionnaires. The mean value of the QuickDASH questionnaire was 12.09 (0–100) for the collagenase group and 8.17 (0–100) for the control group (*p* = 0.157). For the URAM questionnaire, we observed a significantly better result in the control group with 3.46 (0–45) vs. 8.89 (0–45) for the collagenase group (*p* < 0.01).

**Figure 3:**
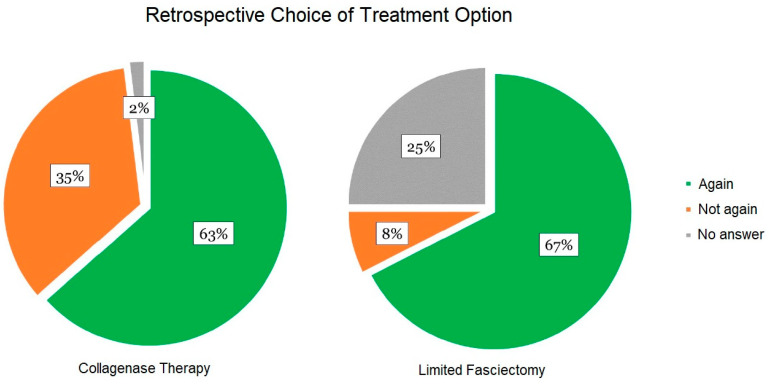
Retrospective decision of patients to choose the performed treatment again (collagenase group n = 52, limited fasciectomy group n = 40). Significantly more patients in the collagenase group would not choose the same treatment again (*p* < 0.05).

The subjective satisfaction of the patients regarding the applied method was inquired about during follow-up examinations (control group, n = 40; collagenase therapy, n = 52). The most frequently mentioned subjective advantages of collagenase therapy were the short duration of therapy (n = 14), avoidance of surgery (n = 11), and the simplicity of the procedure (n = 9). Negative aspects included severe pain during treatment (n = 20), local side effects (n = 6), and high therapy costs, which were not covered by German health care at the time of this study (approximately 1200 €) (n = 5). Overall, 33 out of 52 surveyed patients would retrospectively choose collagenase therapy again (Figure 3). In the case of limited fasciectomy, positive aspects mainly included satisfaction with the long-term results (n = 9) and complete removal of all altered tissue (n = 3). Subjective negative aspects were the necessity of anesthesia (n = 4), hospitalization (n = 3), and lengthy and complex follow-up care (n = 3). Overall, 27 out of 40 patients would retrospectively choose limited fasciectomy as treatment for Dupuytren’s contracture again (Figure 3). Significantly more patients in the collagenase group would not choose the same treatment again when compared to the limited fasciectomy group (*p* < 0.05).

## 4. Discussion

The available data on the long-term effects of collagenase therapy are still limited. Moreover, data comparing the outcomes of collagenase therapy to those of limited fasciectomy treatment are scarce [17,36,37,38,39,40,41,42,43,44,45]. Hupez et al. compared the therapy outcomes between collagenase-treated and surgically treated patients with DD affecting isolated joints after 1 year in a total of 38 patients. For MCP joints, there was no significant difference in residual contracture, but collagenase patients reported lower pain and higher patient satisfaction. The treatment of PIP joints was significantly more successful in patients with surgery, with no differences in patient satisfaction or postintervention pain [45]. Several other studies could verify similar encouraging findings of collagenase treatment in short-term follow-up investigations [46,47,48,49,50]. In the early to mid-postoperative period, many authors observe collagenase is a genuine and cost-effective alternative for the treatment of isolated MCP contractures [44,45,47]. Also, with regard to patient satisfaction, collagenase therapy was not evaluated as inferior to limited fasciectomy in short- or mid-term evaluations [51,52,53].

In long-term evaluations of treatment outcomes with collagenase, these rather optimistic results change. Especially in the PIP joint, a high recurrence rate has been reported [23,48,54]. In the current literature, there are only a few comparative studies on the long-term outcomes of collagenase therapy versus limited fasciectomy that match the sample size and follow-up period of the study presented here [23,24]. The sparse data availability makes our study particularly valuable in evaluating these different therapeutic approaches.

We observed a significant reduction in the Tubiana stage for both collagenase therapy and limited fasciectomy 5.5 and 5.7 years after treatment, respectively (Figure 2). Moreover, the initial Tubiana stage of patients treated surgically was on average significantly more severe than in the collagenase group (2.5 vs. 1.9), while the outcome was better in patients with limited fasciectomy (1.0 vs. 1.2). Accordingly, it seems that more severe contractures can be more effectively treated with surgery. Other studies, including a survey of hand surgeons about the use of collagenase therapy in Dupuytren’s disease, support this conclusion. Indeed, the participants of the survey preferred surgical therapy in more complex cases of Dupuytren’s contracture [23,55].

In minimally invasive techniques such as collagenase treatment, a major complication involves flexor tendon rupture (approximately 0.3%) [43,56,57,58]. While we observed a high frequency of substantial adverse effects directly after intervention, such as swelling, redness, hematoma, pain in the injection area, and skin tears following collagenase treatment, we recorded no long-term adverse effects in our collective, particularly no flexor tendon ruptures [25]. These findings align with those of comparable studies. Arora et al. observed no side effects, particularly no flexor tendon ruptures, after 90 days; other studies show similar results after 2 and 5 years [37,38,42]. The CORDLESS study reported only mild long-term adverse drug effects, specifically skin atrophy around the injected finger [43].

In the control group (limited fasciectomy) we observed two cases (5%) with minor wound healing disorders. This is significantly less than the rate reported by other studies, which report wound healing disorders in 22.9%, infection in 2.4%, nerve injuries in 3.4%, and finger artery lesions in 2% of cases [4,11,59]. This discrepancy may be due to the relatively small patient cohort and/or due to the specialization of the study hospital. However, one case developed a complex regional pain syndrome (2.5%). This is considered as a major complication of the procedure and is estimated to occur with a prevalence of approximately 4.5% [43,56].

We observed no significant difference in grip strength or the pinch test within the groups (Table 3). Moreover, both the treated and untreated fingers of patients with limited fasciectomy had a superior two-point discrimination (*p* < 0.001). As the difference was both on fingers with and without intervention, we attribute this finding to the small patient cohort in this study.

Based on two questionnaires (URAM and QuickDASH), we observed high satisfaction with each therapy in both patient groups, with the surgically treated group outperforming the collagenase group in both questionnaires.

The URAM scale is highly responsive to changes in Dupuytren’s contracture and demonstrates a strong correlation with the progression of contracture, as assessed by the Tubiana scale. In our study, a significant improvement in patient satisfaction was observed in the URAM questionnaire (*p* < 0.01). Conversely, Naam and colleagues reported similar results for the DASH scores in 25 CCH-treated and 21 surgically treated patients after 2 years [39]. Leclère et al. used the Michigan Hand Questionnaire (MHQ) after a 2-year observation period and found high to very high satisfaction in 92% of collagenase-treated and 71% of surgically treated patients [44]. The difference in observation period likely accounts for the discrepancy of our results compared to these findings, as the risk of recurrence of the contracture, in particular after minimally invasive treatments, increases over the years [22,60].. This is likely to affect patient satisfaction in the long term. Indeed, significantly more patients of the collagenase group would not choose the same therapy again when compared to patients with limited fasciectomy (Figure 3).

A benefit assessment of collagenase therapy from 2012 states the average costs of the various therapy options for DD in Germany [61]. Here, collagenase therapy was quantified at 980.09 € (after deduction of legally required discounts) per strand, while surgical limited fasciectomy was reimbursed at 393.41 € for outpatients and 3100.00 € per surgery for inpatients [61]. In line with this, cost-effectiveness studies from Austria, Japan, and the UK have demonstrated that collagenase therapy is more cost-effective than limited partial fasciectomy, primarily due to its lower personnel and material costs, making it a more economical treatment option for Dupuytren’s disease [18,50,54,62,63]. However, the significantly higher long-term recurrence rate for collagenase treatment must also be taken into account. Indeed, we and others previously reported on a high recurrence rate between 39% (MCP joint) and 66% (PIP joint) several years after injection, depending on the treated joint [22,23,24,43,54]. Conversely, in their meta-analysis, Chen and colleagues reported on a recurrence rate after limited fasciectomy between 12% and 31% [12]. From an economic point of view, we therefore see no clear advantage for either therapy option.

This study is a monocentric study conducted in Germany, comparing two therapeutic approaches for DD, with a particular focus on the long-term evaluation of results. We were able to observe a solid number of patients (n = 35 and n = 40) over an above-average period (5.7 and 5.5 years, respectively) and gain valuable insights on the progression of Tubiana stages over time as well as on patient satisfaction and other PROMs. However, some weaknesses of the study can be attributed to its design. The monocentric design can be considered a limitation. Additionally, the study population represents a rather localized group from southern Germany. The relatively small sample size and potential biases during patient recall also represent limiting factors. A multicentric study with a larger study population would have yielded results with significantly higher quality. Moreover, we cannot rule out a selection bias as patients were not randomized for collagenase treatment. Thus, patients with multiple strands and higher stages of DD may have been excluded for treatment with collagenase by the attending surgeon. The significantly higher pre-intervention Tubiana stage in the control group supports this argument (Figure 2). For future research, randomized multicentric trials would be desirable. Additionally, further studies should explore the combination and additive effects of various surgical and non-surgical treatments. Obviously, this research is hindered in Europe by the non-approval of collagenase for the treatment of Dupuytren’s contracture by the European Medicines Agency (EMA).

## 5. Conclusions

In this study we demonstrate that both collagenase therapy and limited fasciectomy are associated with high patient satisfaction and good long-term results. However, significantly more patients in the collagenase group would not choose the same therapy in hindsight (Figure 3). Additionally, we observed a better long-term improvement in the Tubiana stage in the group with limited fasciectomy (Figure 2). While more and extensive studies are necessary to allow for a definitive recommendation, it seems most likely that the two procedures should not be seen as two distinct therapy options but rather as complementary for the treatment of DD. Due to the low incidence of long-term adverse effects, collagenase therapy seems a sensible option for DD with a lower Tubiana stage and contractures that predominantly affect the MCP joint. Dupuytren’s contractures with higher Tubiana stages that also affect the PIP joint should predominantly be treated with limited fasciectomy.

## Figures and Tables

**Table 1:** Subjective treatment parameters assessed during follow-up examination.

Parameter	Possible Response
Pain and evaluation of therapy choice	
Subjective pain in the treated hand Subjective positive and negative aspects of the treatment In retrospect: same choice of therapy	yes/no open question yes/no

**Table 2:** Characteristics of patient cohort.

	Collagenase *	Limited Fasciectomy
Total	35	40
Male	28	37
Female	7	3
Age at the time of treatment (Mean ± SD)	68 (8.4)	67 (8.7)
Affected digits		
Total	41	69
Middle finger	5	7
Ring finger	21	27
Little finger	15	32

In the collagenase group the following immediate adverse effects were observed: pain in the injection area (n = 4), swelling (n = 20), skin tear (n = 11), hematoma (n = 20), redness (n = 9), and isolated, mild side-effects (n = 6) [25]. We observed no residual symptoms of these side-effects in 5.5 years after infiltration. Long-term adverse effects on the control group were one case of CRPS and wound healing disorders in two patients that were treated without additional surgery. * Data from the collagenase group previously published by our research team [22].

**Table 3:** Grip strength and pinch test measurements in the long-term follow-up (after 5.5 and 5.7 years, respectively) of the two patient groups.

		Collagenase	Limited Fasciectomy
Grip Strength	Treated (kg ± SD)	36.6 (11.4)	35.3 (8.8)
	Untreated (kg ± SD)	34.8 (11.1)	34.9 (8.8)
	Significance of difference between sides (*p*)	0.079	0.571
Pinch Test	Treated (kg ± SD)	3.2 (1.8)	3.1 (1.8)
	Untreated (kg ± SD)	3.0 (1.5)	3.1 (1.6)
	Significance of difference between sides (*p*)	0.339	0.917

## Data Availability

The original contributions presented in the study are included in the article.
